# Tobacco-Free Cigarette Smoke Exposure Induces Anxiety and Panic-Related Behaviours in Male Wistar Rats

**DOI:** 10.1038/s41598-018-23425-z

**Published:** 2018-03-21

**Authors:** Máira Tereza Talma Chírico, Frank Silva Bezerra, Mariana Reis Guedes, Ana Beatriz Souza, Fernanda Cacilda Silva, Glenda Campos, Sylvana Rendeiro de Noronha, Laura Batista Tavares Mesquita, Thayane Oliveira Reis, Silvia Dantas Cangussú, Deoclécio Alves Chianca-Jr, Rodrigo Cunha de Menezes

**Affiliations:** 1 Department of Biological Sciences, Institute of Exact and Biological Sciences, Federal University of Ouro Preto, Ouro Preto, MG Brazil; 2 Graduate Program in Biological Sciences – CBIOL/NUPEB, Federal University of Ouro Preto, Ouro Preto, Brazil

## Abstract

Smokers, who generally present with lung damage, are more anxious than non-smokers and have an associated augmented risk of panic. Considering that lung damage signals specific neural pathways that are related to affective responses, the aim of the present study was to evaluate the influence of pulmonary injury on anxiety and panic-like behaviours in animals exposed to cigarette smoke with and without tobacco. Male Wistar rats were divided into the following groups: a control group (CG); a regular cigarette group (RC); and a tobacco-free cigarette (TFC) group. Animals were exposed to twelve cigarettes per day for eight consecutive days. The animals were then exposed to an elevated T-maze and an open field. The RC and TFC groups presented increases in inflammatory cell inflow, antioxidant enzyme activity, and TBARS levels, and a decrease in the GSH/GSSG ratio was observed in the TFC group. Exposure to RC smoke reduced anxiety and panic-related behaviours. On the other hand, TFC induced anxiety and panic-related behaviours. Thus, our results contradict the concept that nicotine is solely accountable for shifted behavioural patterns caused by smoking, in that exposure to TFC smoke causes anxiety and panic-related behaviours.

## Introduction

Cigarette smoke exposure is associated with anxiety states. Smokers are more anxious than non-smokers^1^, while cigarette smoking cessation is associated with increased levels of anxiety and stress, as the nicotine in cigarettes has been shown to have anxiolytic effects^2^. Moreover, smoking is also associated with an augmented risk of panic attacks, and quitting smoking could help reduce this risk^3^. Importantly, in a study conducted by Amaring and colleagues, it was reported that 72% of panic disorder patients declared that they were regular smokers at the onset of their disease^4^.

Cigarette smoke is also one of the several agents and environmental factors that can trigger oxidative stress and pulmonary damage^5^. Cigarette smoke causes cellular recruitment, lipid peroxidation, production of inflammatory mediators, and oxidative stress^6–11^. For instance, studies in mice have shown that exposure to short-term cigarette smoke evokes an increase in inflammatory cell inflow and oxidative damage^6,9^. In general, exposure to pollutants induces pulmonary inflammation through the generation of oxidative stress^12,13^, defined as the imbalance in reactive oxygen species production, to the detriment of the antioxidant defence systems^14^. Importantly, exposure to ambient air particles not only induces pulmonary inflammation but also behavioural disorders both in humans and in mice^15^.

Currently, the majority of anxiety studies associated with cigarette smoking have focused on the anxiolytic effects of nicotine^2^. However, it has been shown that lung damage can induce central nervous system responses by activating specific neuronal pathways^16,17^, which include those linked to affective responses, such as anxiety and panic^18^. This raises the question of whether the anxiety and panic-type behaviour associated with smoking might be related not only to the nicotine or to tobacco’s other constituents but also to lung damage.

The purpose of this study was to investigate the effect of smoke exposure, and therefore, of lung damage, produced by regular and tobacco-free cigarettes (TFC) on anxiety and panic-associated behaviours in male Wistar rats. As regular cigarettes are known to contain anxiolytic substances, such as nicotine^19^, we used TFC in order to evaluate the effect of smoke and the corresponding lung damage caused by it, on anxiety and panic-related behaviours without the influence of tobacco. To this end, we compared the influence of exposure to the smoke produced by these two cigarette types on lung damage by analysing inflammatory cell influx and oxidative stress and the effect on the aforementioned behaviours by using the elevated T-maze paradigm.

## Results

### Tobacco-free and regular cigarette smoke exposure produce similar effects on leucocyte influx but not on the BALF inflammatory cell profile

**Figure 1:**
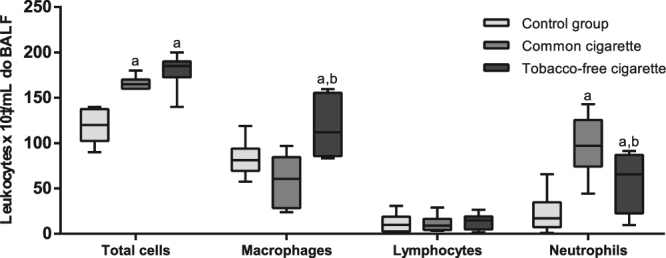
Cigarette smoke exposure effects on leukocyte influx and BALF inflammatory cell profile. Letters (above the bars) indicate significant differences (a), difference in relation to the control group; (b), difference between the cigarette groups) analysed by one-way ANOVA, followed by a Newman Keuls post hoc test (p < 0.05). Data are expressed as the mean ± SEM (n = 8 in each group).

Smoke exposure to both TFC and RC increased the inflammatory cell influx to lung parenchyma, reflected in a higher BALF leucocyte cell count, compared to the control group [F (2, 21) = 32.89; p < 0.0001]. The macrophage count in animals exposed to TFC smoke was, on average, 41% higher than that of the control animals and 42% higher than that of animals exposed to RC smoke. The neutrophil count in animals exposed to RC smoke was increased by 410% compared to the CG and by 63% compared to the TFC group, on average. Furthermore, the TFC group was 251% higher than that of the CG, on average. The lymphocyte counts did not present differences (Fig. 1).

### Regular cigarette smoke exposure increased the volume density of the alveolar space and reduced the alveolar septa

**Figure 2:**
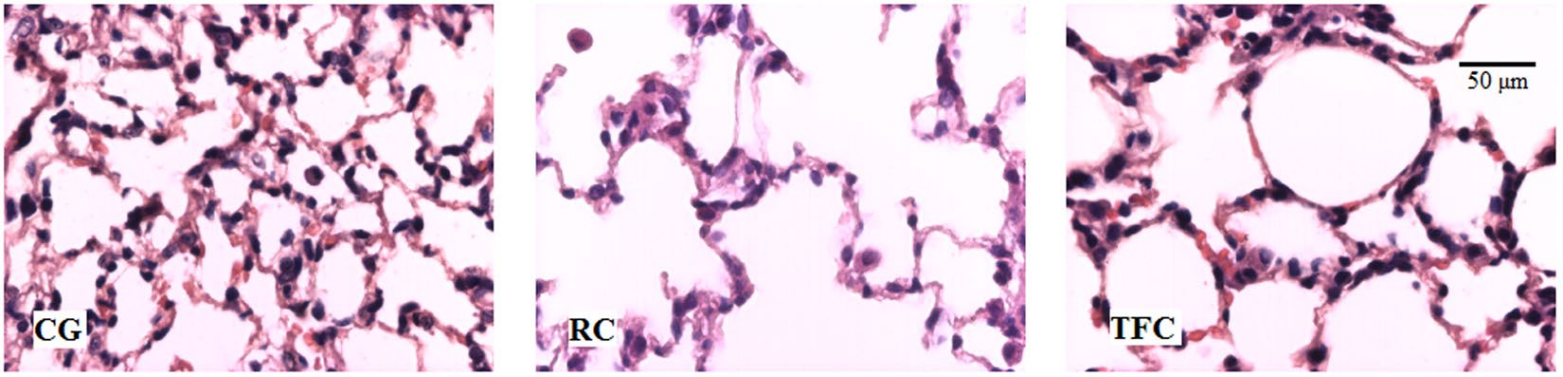
Illustrations of the increased alveolar space and decreased alveolar septal volume in lung section photomicrographs of the animals exposed to cigarette smoke. CG: control group; RC: regular cigarette; TFC: tobacco-free cigarette. 40x magnification.

The (Vv [a]) of the lung parenchyma increased in response to exposure to RC compared to the controls (p = 0.0078) (Table 1). The (Vv [sa]) of the animals exposed the RC smoke was smaller than that of the controls (p = 0.0120). Lung photomicrograph sections illustrate these results (Fig. 2).

**Table 1:** Cigarette smoke increased the volume density of the alveolar space and decreased the alveolar septa in histological lung sections. Letters indicate significant differences ^a^difference in relation to the control group) analysed by the Kruskal-Wallis test, followed by Dunn’s post-test (p < 0.05). Data are given as median, minimum, and maximum values (n = 8). (Vv [a]): alveolar spaces; (Vv [sa]): alveolar septa. CG: control group; RC: regular cigarette; TFC: tobacco-free cigarette.

Stereology	Groups		
	CG	RC	TFC
Vv[a](%/mm²)	43.60 (26.56–55.31)	55.94 (51.25–64.38)^a^	51.56 (47.81–61.25)
Vv[sa](%/mm²)	55.47 (43.44–72.19)	43.13 (35.00–48.75)^a^	48.29 (38.13–51.88)

### Differential effect of tobacco-free and regular cigarette smoke exposure on oxidative stress biomarkers and antioxidant defences in the lung

The levels of TBARS increased in both groups exposed to the cigarette smoke (RC and TFC) in relation to controls [F (2, 17) = 8.13; p = 0.0033]. The activity of both the antioxidant enzyme SOD [F (2, 21) = 10.29; p = 0.0008] and CAT [F (2, 21) = 12.53; p = 0.0003] increased in animals exposed to RC and TFC smoke compared to controls. The glutathione levels, evaluated by the ratio of GSH/GSSG, were smaller in TFC animals compared to control animals (p = 0.0143). The carbonyl protein content was higher in animals exposed to tobacco-free cigarette smoke compared to the CG [F (2, 21) = 6.27; p = 0.0073] (Table 2).

**Table 2:** Effects of exposure to tobacco-free or regular cigarette smoke on the activity of antioxidant enzymes (SOD and CAT), the GSH/GSSG ratio, TBARS, and carbonyl protein) in lung samples. Letters above the bars indicate significant differences between the groups (^a^difference in relation to the control group; ^b^difference between the cigarette groups) analysed by one-way ANOVA, followed by a Newman Keuls post-test (p < 0.05). Data are expressed as the mean ± SEM (n = 8). CG: control group; RC: regular cigarette; TFC: tobacco-free cigarette; TBARS: thiobarbituric acid-reactive substances; SOD: superoxide dismutase; CAT: catalase; GSH: reduced glutathione; GSSG: oxidized glutathione.

	CG	RC	TFC	p*
TBARS (nM/mg prot)	1.00 ± 0.07	1.57 ± 0.14^a^	1.54 ± 0.10^a^	0.0033
SOD (U/mg prot)	25.73 ± 1.09	34.90 ± 1.86^a^	40.65 ± 3.44^a^	0,0008
CAT (U/mg prot)	0.48 ± 0.06	0.96 ± 0.06^a^	0.94 ± 0.10^a^	0.0003
GSH	79.56 ± 7.52	29.14 ± 2.26^a^	42.08 ± 14.23^a^	0.0037
GSSG	10.96 ± 0.74	6.85 ± 0.57^a^	11.80 ± 1.85^b^	0.0164
GSH / GSSG ratio	7.04 ± 0.55	4.50 ± 0.66	2.94 ± 0.82^a^	0.0143
Carbonyl protein (nmol/mg)	9.93 ± 0.75	7.07 ± 0.81	12.88 ± 1.68^b^	0.0073

### Effects of cigarette smoke on anxiety and panic-related behaviours

#### Tobacco-free cigarette smoke exposure leads to anxiety-related behaviour, while regular cigarette smoke has an anxiolytic effect

**Figure 3:**
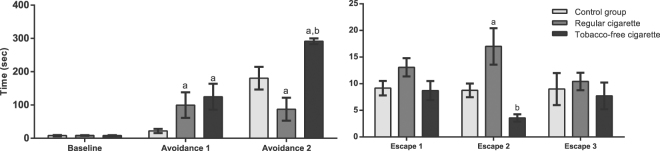
Effects of cigarette smoke exposure on the ETM. (a) Time spent by the animals in leaving the ETM’s enclosed arm during baseline, avoidance 1, and avoidance 2 over three trials (with 30-s intervals and a cut-off time of 300 s). (b) Time spent by the animals in leaving the ETM’s open arm during escape 1–3 over three trials (with 30-s intervals and a cut-off time of 300 s). Letters above the bars indicate significant differences between the groups (a), difference in relation to the control group; (b), difference between the cigarette groups) analysed by one-way and two-way ANOVA, followed by Newman Keuls post hoc tests (p < 0.05). Data are expressed as the mean ± SEM (n = 8–12 in each group).

The influence of regular cigarettes and tobacco-free cigarettes on anxiety-related behaviour was evaluated using the ETM. Two-way ANOVA revealed effects of treatment [F (2, 29) = 3.79; p = 0.0344], trials [F (2, 58 = 46.06; p < 0.0001), and of the trials by treatment interaction [F (4, 58) = 7.84; p < 0.0001] on inhibitory avoidance (Fig. 3A). The post hoc test showed that smoke exposure to both tobacco-free cigarettes and commercial cigarettes reduced the avoidance 1 latency (p < 0.005) compared to the controls. Regarding avoidance 2, the regular cigarette group exhibited reduced latency in the closed arm in relation to the control group (p < 0.005), demonstrating an anxiolytic effect. However, the tobacco-free cigarette group exhibited an increased latency to leave the closed arm compared to the control and regular cigarette smoke groups (p < 0.005), demonstrating an anxiogenic affect.

The increased latency to leave the closed arm on avoidance 2 was positively correlated, when the data from the control and TFC-exposed rats were analysed together, with increases in the levels of SOD (r = 0.64, p = 0.01) and TBARS (r = 0.66, p = 0.0074) and in the number of cells in BALF (r = 0.55, p = 0.035). Moreover, when the data from rats exposed to regular cigarettes (which led to an anxiolytic effect) were included in the analysis there was no correlation between any variable and the behavioural output, as expected [SOD (r = 0.6374, p = 0.07), TBARS (r = 0.32, p = 0.13) and in the number of cells in the BALF (r = 0.21, p = 0.31)].

#### Tobacco-free cigarette smoke exposure leads to panic-related behaviour, while regular cigarette smoke has a panicolytic effect

The influence of regular cigarettes and tobacco-free cigarettes on panic-related behaviour was evaluated using the ETM. Two-way ANOVA revealed the effects of treatment [F (2, 28) = 4.77; p = 0.0165] (Fig. 3B). However, there was no significant difference in the interaction or regarding the trials. The post hoc test showed that, on the second attempt, the animals exposed to regular cigarette smoke exhibited an increased latency in the open arm compared to the control animals (p < 0.005), demonstrating a panicolytic effect. On the other hand, the group exposed to paper cigarette smoke exhibited a reduced latency to leave the open arm compared to the group exposed to regular cigarette smoke (p < 0.005), demonstrating a panicogenic effect. The increased latency to leave the open arm on escape 2 was negatively correlated, when data from the control and TFC-exposed rats were analysed together, with increases in the levels of SOD (r = −0.54, p = 0.03) and catalase (r = −0.60, p = 0.018), TBARS (r = −0.58, p = 0.02) and in the number of neutrophils in the BALF (r = −0.54, p = 0.036). Moreover, when the data from the rats exposed to regular cigarettes (which led to an anxiolytic effect) was included in the analyses, there was no correlation between any variable and the behavioural output, as expected [SOD (r = 0.04, p = 0.83), catalase (r = 0.16, p = 0.46), TBARS (r = −0.05, p = 0.9), or the number of neutrophils in the BALF (r = 0.39, p = 0.06)].

### Differential locomotor activity of tobacco-free and regular cigarette smoke exposure

**Figure 4:**
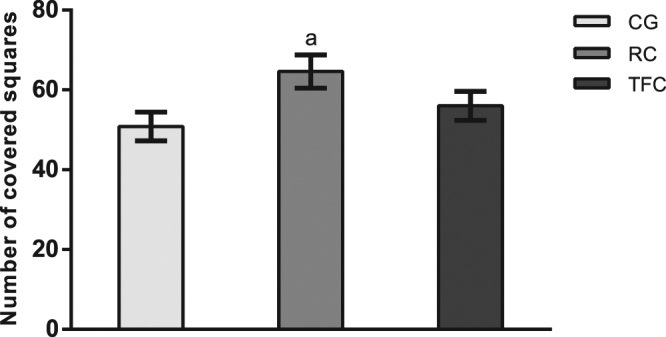
Cigarette smoke effect on locomotor activity. Letters above the bars indicate significant differences between the groups (a) difference in relation to the control group; (b) difference between the cigarette groups) analysed by one-way and two-way ANOVA, followed by Newman Keuls post hoc tests (p < 0.05). Data are expressed as the mean ± SEM (n = 8–12 in each group). CG: control group; RC: regular cigarette; TFC: tobacco-free cigarette.

The locomotor activity was higher in the group exposed to the regular cigarettes in relation to the control group [F (2, 30) = 3.53; p = 0.0422] (Fig. 4). However, the animals exposed to TFC smoke had similar locomotor activity compared to the control animals.

## Discussion

In this study, we showed that cigarette smoke exposure, even without the presence of tobacco, promoted lung inflammatory injury, as indicated by the presence of inflammatory cells in BALF and oxidative stress (measured by TBARS levels, a reduced GSH/GSSG ratio, and increased activity of antioxidant enzymes – SOD and CAT). Moreover, whereas exposure to the regular cigarette smoke (with tobacco) reduced anxiety and panic-type behaviour due to the presence of nicotine and other components, the tobacco-free cigarette smoke promoted anxiety and panic-related behaviours. Notably, these behavioural changes were correlated with increases in oxidative stress markers and cell migration to the lung. Considering the demonstrated anxiolytic effect of tobacco^20^, these results suggest that anxiety related to smoking may result, at least in part, from the lung damage caused by smoke components.

Previous results^6,7,10,11,21–23^ have already shown that regular cigarette smoke promotes an increase in bronchoalveolar lavage inflammatory cellular influx. The present study showed that such an effect cannot be solely attributed to tobacco and its components, as tobacco-free cigarette smoke promoted inflammatory cellular inflow in the bronchoalveolar lavage, suggesting the presence of a local inflammatory process. Regarding the cell count differences, there was a macrophage reduction and a neutrophil increase in the RC group. In the TFC group, there was an increase in these cell types. These data suggested a lung inflammatory process in both groups compared to the control group.

The pulmonary parenchyma histoarchitecture analysis showed an increase in the volume density of alveolar spaces and a reduction in the volume density of the alveolar septa in animals exposed to regular cigarette smoke compared to control animals, suggesting lung parenchyma damage. Interestingly, the tobacco-free smoke did not change the lung histoarchitecture, suggesting that smoke produced by tobacco is more detrimental to the lung.

Beyond inflammatory cell influx and pulmonary histoarchitecture, it was also important to evaluate oxidative stress and antioxidant enzyme activity during the smoke exposure process. Lipid oxidation is broadly evaluated by the TBARS assay^24^. It is not a novel observation that TBARS levels are increased in animals exposed to regular cigarette smoke^10,25,26^. Nevertheless, to our knowledge, this is the first time that tobacco-free cigarette smoke has been found to be able to induce pulmonary parenchymal lipid peroxidation. Additionally, the carbonyl protein concentration (another way to measure protein membrane damage in the pulmonary parenchyma) was higher in the TFC group than in the RC group. The GSH/GSSG ratio is an important marker of oxidative stress^27^. Studies have already shown that regular cigarette smoke (with tobacco) reduces the GSH/GSSG ratio^28,29^. Here, we showed that exposure to the smoke of a tobacco-free cigarette decreases the GSH/GSSG ratio in relation to the control, confirming that the tobacco-free cigarette causes oxidative stress in the lung. We also analysed the activity of two antioxidant enzymes, which are crucial in the oxidant/antioxidant balance. CAT and SOD are considered the most important antioxidant enzymes in the airways^30,31^. Exposure to TFC smoke caused an increase in antioxidant enzyme activity (SOD and CAT), as has been previously reported in response to RC smoke^11,23^. Thus, when faced with exposure to smoke, the body promotes an increase in SOD and CAT activity to try to reverse this oxidative damage, further avoiding the formation of the hydroxyl radical, which does not have a defence system against it^32^.

Exposure to regular cigarette smoke reduced anxiety and panic-related behaviours as assessed by the elevated T-maze. The nicotine effects of cigarette smoke exposure are relevant^33^; however, evidence has suggested that nicotine is not the only component of the cigarette that influences behaviour. Other components of tobacco smoke may also play a role in these changes^20^. In the present study, animals exposed to TFC smoke (without tobacco) presented anxiety-related behaviour in the elevated T-maze. Importantly, the anxiogenic responses observed in these animals were positively correlated with increases in SOD, TBARS and the total number of inflammatory cells in the BALF, suggesting that lung damage could be important in generating signals that could lead to anxiety-related behaviours. Moreover, when the lung damage was paired with the anxiolytic substances present in the tobacco, such as nicotine, these correlations disappeared, strengthening the case that this lung damage can lead to anxiety-related behaviours. Recent studies have proposed that smoking increases stress in smokers rather than decreasing it, as previously thought, and that the period after smoking cessation immediately generates anxiety due to nicotine withdrawal and not smoking itself^34,35^. However, our results lead us to interpret these changes differently since we showed that the tobacco-free cigarettes, beyond causing lung damage, also promote anxiety and panic-related behaviours. Interestingly, exposure to air pollutants, which is known to lead to lung damage, leads to anxiety-related behaviour and depression-like behaviours in mice and leads to mood disorders in humans^15^.

Animals exposed to TFC smoke also presented panic-related behaviour in the elevated T-maze. Importantly, the panicogenic responses observed in these animals were positively correlated with increases in SOD, TBARS and the number of neutrophils in the BALF, suggesting that lung damage could also generate signals that could lead to panic-related behaviours. Moreover, when the lung damage was paired with tobacco, these correlations disappeared, indicating that lung damage can lead to panic-related behaviours. In humans, smoking have been clearly associated with the onset of panic disorders^3,4^. Moreover, smokers with asthma, which also increases the risk of panic attacks^36^, have greater levels of anxiety and panic symptoms and are at an increased risk for a lifetime history of panic attacks compared to smokers without asthma^37^. We, therefore, suggest that lung damage, or inflammation, may be directly involved in the behavioural changes caused by exposure to smoke, either from cigarettes, pollution or even asthma.

After the elevated T-maze test, the locomotor activity was evaluated in the open field test in order to validate and confirm the behavioural responses seen in the apparatus^38^. Our findings indicated that the behaviour (anxiety and panic-related) of both groups (RC and TFC) was not induced by alterations in locomotor activity.

Thus, this work provided new insights about the strong relationship between smoke exposure and behavioural patterns, where the changes caused by smoke, such as inflammation and oxidative stress, could lead to anxiety and panic-related behaviours, independent of the influence of nicotine or other tobacco components. Our results contradicted the concept that tobacco is solely accountable for shifted behavioural patterns caused by smoking.

## Methods

### Animals

Male Wistar rats (180 ± 10 g) were randomly divided into three groups: regular and tobacco-free cigarettes groups and a control group (n = 8–12/group). Four animals were housed per cage. The rats received water and food *ad libitum* and remained under a controlled temperature (23 ± 1 °C) and a dark/light cycle (12:12 h). All procedures and the experimental design were approved by the Ethical Committee for Animal Research of the Federal University of Ouro Preto (Protocol n° 2015/35), according to the Brazilian Society of Neuroscience and Behaviour and the “National Institutes of Health Guidelines for the Care and Use of Laboratory Animals” (8th edition, 2011).

### Exposure to cigarette smoke

#### Regular cigarette

Filtrate Marlboro^TM^ (10 mg tar, 0.8 mg of nicotine and 10 mg of carbon monoxide) was used as the regular cigarette (RC) in this study. Rats were exposed to twelve cigarettes per day over three periods (morning/afternoon/evening) for eight consecutive days. The number of cigarettes that each rat was exposed to in the present study was based on previous studies^9,25,39^. To perform the cigarette smoke inhalation, a lit cigarette was linked to a plastic syringe (60 mL), which was used to aspirate the smoke. Then, after the cigarette was detached, the syringe was fitted into an inhalation chamber (40 cm length, 30 cm wide, and 25 cm high) to perform the expulsion of the smoke. The cigarette was burned until the final third; this required approximately 27 puffs (insufflation and deflation of the syringe). The estimated time to burn a cigarette was three minutes; however, the animals were exposed to the smoke of each cigarette for six minutes. The chamber cover was removed after this period. Then, the animals were exposed to ambient air for one minute. This procedure was repeated with the remaining cigarettes^9^. All animals were exposed to the smoke at the same time. The control group (CG) was exposed to the same procedures, with the only difference being that the smoke was not infused into the chamber.

#### Tobacco-free cigarette

The tobacco-free cigarette (TFC) consisted of Marlboro filtrate without the tobacco, which was removed and replaced by eight cigarette paper sheets (Cigarette sheet: Trevo^TM^, manufactured especially for Souza Cruz; 76 mm long, 46 mm wide). The total number of cigarette sheets needed for each paper cigarette was determined before the study. To determine the number of sheets to be used in the cigarette without tobacco, we weighed the tobacco in the RC and then replaced it with the number of sheets that would match the weight of the tobacco. We then adjusted the number of sheets to match the time that the RC took to burn (3 min) and the number of puffs (27) produced. The TFC exposure protocol that was performed was identical to the one used for RC exposure.

### Behavioural test

All animals were exposed to behavioural tests to evaluate the influence of cigarette smoke exposure on behavioural patterns. For this, we used the elevated T-maze (ETM), which is an apparatus consisting of two open arms (1 cm Plexiglas rims) and one closed arm (40 cm high wall) of equal dimensions (50 cm × 12 cm) at a distance of 50 cm from the floor. Exposure to the ETM enabled the performance of inhibitory avoidance and escape tests to evaluate anxiety and panic-related behaviours, respectively.

We used the open field (OF) to evaluate locomotor activity. This apparatus consisted of a polypropylene square arena (40 cm long × 32 cm high), whose floor was subdivided into 16 squares and whose walls prevented the animals from exiting.

The ETM and OF were located in an isolated, silent, and illuminated room (60 lux), with the temperature set at 23 ± 1 °C.

### Experimental design

As described above, the animals were exposed to cigarette smoke for eight days (three times a day). On the sixth and the seventh days of cigarette smoke exposure (i.e., on the two days preceding the behavioural tests), the experimenter handled each animal for 5 min to avoid any handling stress on the test day. On the seventh day, the animals were pre-exposed to one of the elevated T-maze open arms for 30 minutes. On this occasion, the open arm was isolated using a wooden board. Such pre-exposure is a standard procedure to guarantee habituation to the exploratory component of the animal’s behaviour in the open arm, thereby leaving only the aversive component to be evaluated during the first exposure to the maze’s open arm^40^.

On the eighth day, the animals were exposed to the ETM three hours after cigarette smoke exposure in order to evaluate them under the influence of the substances present in regular cigarettes and tobacco-free cigarettes. First, each animal was carefully removed from the box and placed at the end of the closed arm (away from the exit). The time spent by the animal to completely get out of the closed arm with all four paws was registered. Each animal was exposed to the closed arm for three trials with a thirty-second interval between them. The first trial was called baseline, the second avoidance 1, and the third avoidance 2. After thirty seconds, each animal was exposed to the open arm for three trials with a thirty-second interval, called escape 1–3. Once again, the time spent by the animal to completely get out of the open arm with all four paws was registered in each trial. The cut-off time for each trial was set at 300 seconds. Thirty seconds after the ETM exposure, the animals were exposed to the OF for 300 seconds. The number of squares traversed by each rat during this time interval was quantified and annotated to evaluate locomotion^41^. Moreover, the OF test was used to validate and confirm the behavioural response observed in the apparatus^38^.

### Bronchoalveolar lavage fluid (BALF) analysis

On the tenth day after commencement of the cigarette smoke exposure (twenty-four hours after the last cigarette smoke exposure), the rats were deeply anaesthetized (80 mg/kg^−1^ ketamine and 11.5 mg/kg^−1^ xylazine, i.p). Next, the trachea was exposed and cannulated (PE90 tubing) and then the right lung was washed with 3 mL of saline (NaCl-0.9%) in order to collect the BALF. Samples were kept on ice until the end of the procedure to avoid cell lysis. The total leukocyte count in the BALF was marked in a Neubauer chamber with trypan blue dye (0.2%). The differential cell counts were performed in a Cytospin centrifuge (Shandon, Waltham, MA, USA). The samples were individually placed on slides at 1,000 rpm for 1 minute. After centrifugation, the lung blades were stained with a Panotic Quick kit (Labourclin, Pinhais, Paraná) for later microscopic analysis^9^.

### Lung analysis

After collecting the BALF, each animal’s thorax was opened to perform a right ventricle perfusion with saline (40 mL, NaCl-0.9%). The left lung was collected and immersed in formalin for 48 hours. Subsequently, it was processed and stained with haematoxylin and eosin for posterior histological analysis. The right lung was removed and stored at −80 °C.

### Morphometric analysis of lung

A test system comprising 16 points in a known test area was used to analyse the volume density (Vv) of the alveolar septa (Vv[sa]) and of the alveolar spaces (Vv[a]). The total number of points of the test system (Pt) was considered as the standard for evaluating the number of points (Pp) coinciding with the (Vv[sa]) and the (Vv[a]). For the analysis, we used a cycloid test system attached to the monitor screen. We analysed 20 photographs (different areas) of each lung blade. Afterwards, we computed the median to reduce bias. We analysed a total area of 1.94 mm^2^ to determine the volume densities of the (Vv[sa]) and the (Vv[a])^42^.

### Oxidative stress biomarkers and antioxidant defences

The CAT activity was measured (absorbance of 240 nm) by the speed with which H_2_O was reduced by the enzyme action^43^. The SOD activity was evaluated (absorbance of 570 nm) via the enzyme’s capacity to inhibit pyrogallol autoxidation^44^. The thiobarbituric acid-reactive substances (TBARS) assay was used to evaluate (absorbance of 535 nm) lipid peroxidation^45^. The carbonyl protein determination method (absorbance of 370 nm) used in this study was based on that described by Reznick^46^. The total glutathione, found by adding the reduced (GSH) and oxidized (GSSG) glutathione, was measured using a commercial kit assay (#CS0260, Sigma-Aldrich Co) and was enzymatically determined (absorbance of 412 nm) using the Griffith assay^47^. The total protein was quantified according to the Bradford method^48^.

### Statistical analysis

The avoidance and escape analyses of the ETM data were performed using repeated measures ANOVA, considering the treatment (RC, TFC or control) as the independent factor and the trial (baseline, avoidance 1 and 2 or escape 1–3) as the dependent factor. A Newman Keuls test was used post hoc for parametric data. Data from the OF, BALF analysis, antioxidant enzymes, TBARS, and carbonyl protein were entered into one-way ANOVA, and a Newman Keuls test was used post hoc. A Kruskal-Wallis test, followed by Dunn’s post-test, was used for discrete data, and these data were expressed as median, minimum, and maximum values. We used p < 0.05 as the threshold for significant values, and each variable was assessed using the Kolmogorov-Smirnov normality test. Pearson correlations were used to quantify the relationships among the pulmonary oxidative markers, cell migration, and the behavioural outputs. All analyses were performed using the GraphPad Prism software version 7.00 (GraphPad, San Diego, CA, USA).

### Data availability

The datasets generated during and/or analysed during the current study are available from the corresponding author upon reasonable request.
